# Supplementary material for: Novel endornaviruses infecting Phytophthora cactorum that attenuate vegetative growth, promote sporangia formation and confer hypervirulence to the host oomycete
Source: J Gen Virol. 2025 May 1;106(5):002099. doi: 10.1099/jgv.0.002099 (PMC12046096; doi:10.1099/jgv.0.002099)
Supplement: Uncited Supplementary Material 1. [file jgv-106-02099-s001.pdf]

Amino acid(similarity)

Nucleotide

	PcAEV1	PcAEV2	PcAEV3	PcAEV4	PcAEV5
PcAEV1		20.1%(35.0%)	19.3%(33.8%)	19.9%(34.0%)	19.2%(32.5%)
PcAEV2	44.8%		44.6%(63.0 %)	58.4%(75.4 %)	44.4%(63.3 %)
PcAEV3	44.6%	54.0%		44.8%(63.1 %)	83.7%(91.9 %)
PcAEV4	44.6%	59.6%	53.4%		45.7%(63.7 %)
PcAEV5	44.3%	54.0%	73.2%	54.6%	

~ 20 %	21 ~ 40 %	41 ~ 60 %	61 ~ 80 %	81 % ~

Supplemental Figure S1. Pairwise sequence alignment (PSA) comparing the complete nucleotide and amino acid sequences of PcAEV1-5. PSA was performed using EMBOSS Needle, and the identities (similarities in parentheses) of the complete nucleotide and amino acid sequences are shown. To clarify the results, darker blue indicates higher percentages, with 100% identity removed by the diagonal line.

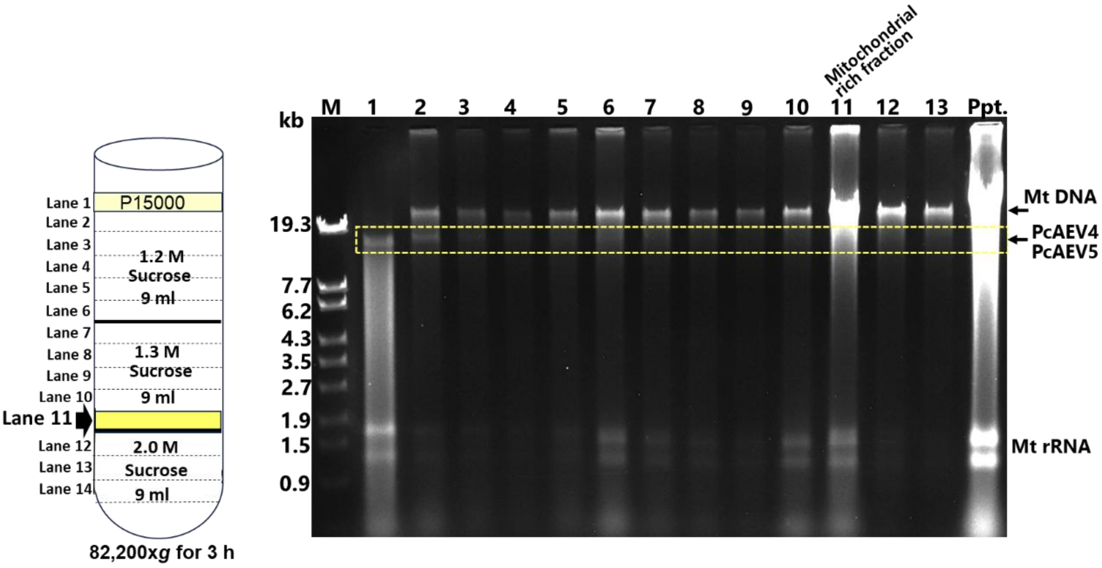

Supplemental Figure S2. Discontinuous sucrose gradient fractionation of crude mitochondrial fraction P15000. Fractions obtained were labeled 1 through 14 from top to bottom (Lanes 1-14). Total nucleic acids were extracted from 1/10 of each fraction and subjected to agarose gel electrophoresis. Lane M: DNA marker (250 ng of λDNA digested with EcoT14I). The positions of PcAEV4 and PcAEV5 dsRNA are indicated by yellow dashed lines and arrows. Mt DNA: mitochondrial genomic DNA; Mt rRNA: mitochondrial ribosomal RNA.

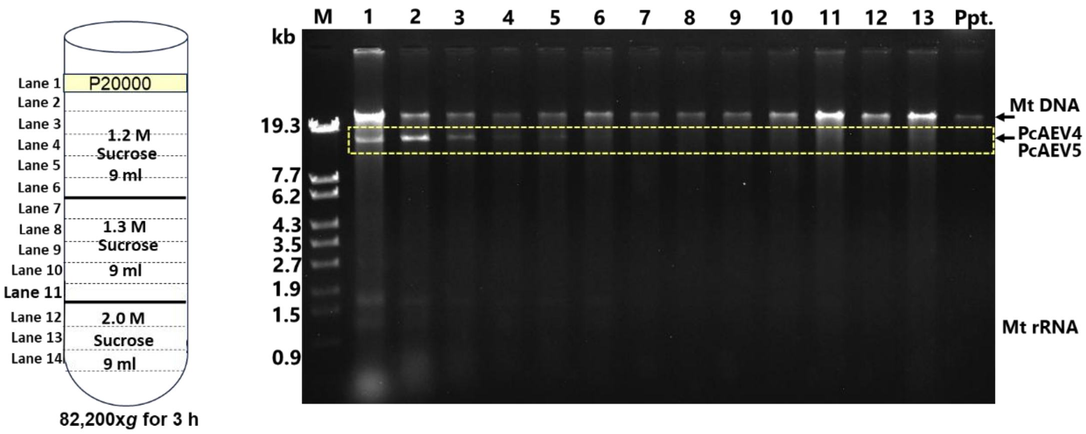

Supplemental Figure S3. Discontinuous sucrose gradient fractionation of P20000. Fractions obtained were labeled 1 through 14 from top to bottom (Lanes 1-14). Total nucleic acids were extracted from 1/10 of each fraction and subjected to agarose gel electrophoresis. Lane M: DNA marker (250 ng of λDNA digested with EcoT14I). The positions of PcAEV4 and PcAEV5 dsRNA are indicated by yellow dashed lines and arrows. Mt DNA: mitochondrial genomic DNA; Mt rRNA: mitochondrial ribosomal RNA.

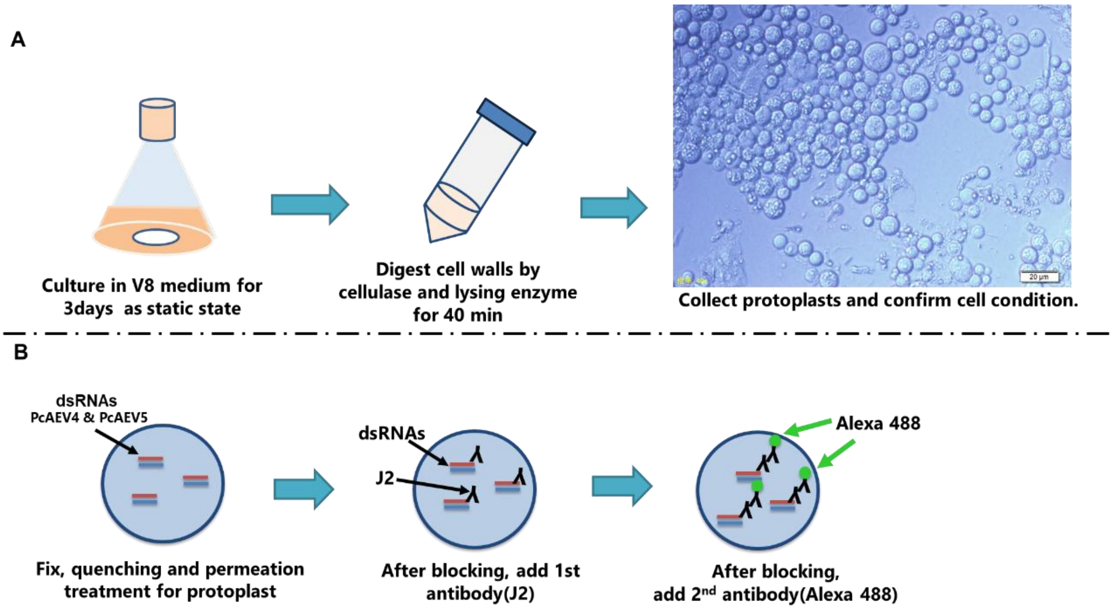

Supplemental Figure S4. IFA scheme for detecting dsRNA in *Phytophthora* spp. cells. (A) Schematic of protoplast preparation for IFA. **(B)** Schematic of IFA in protoplasts.

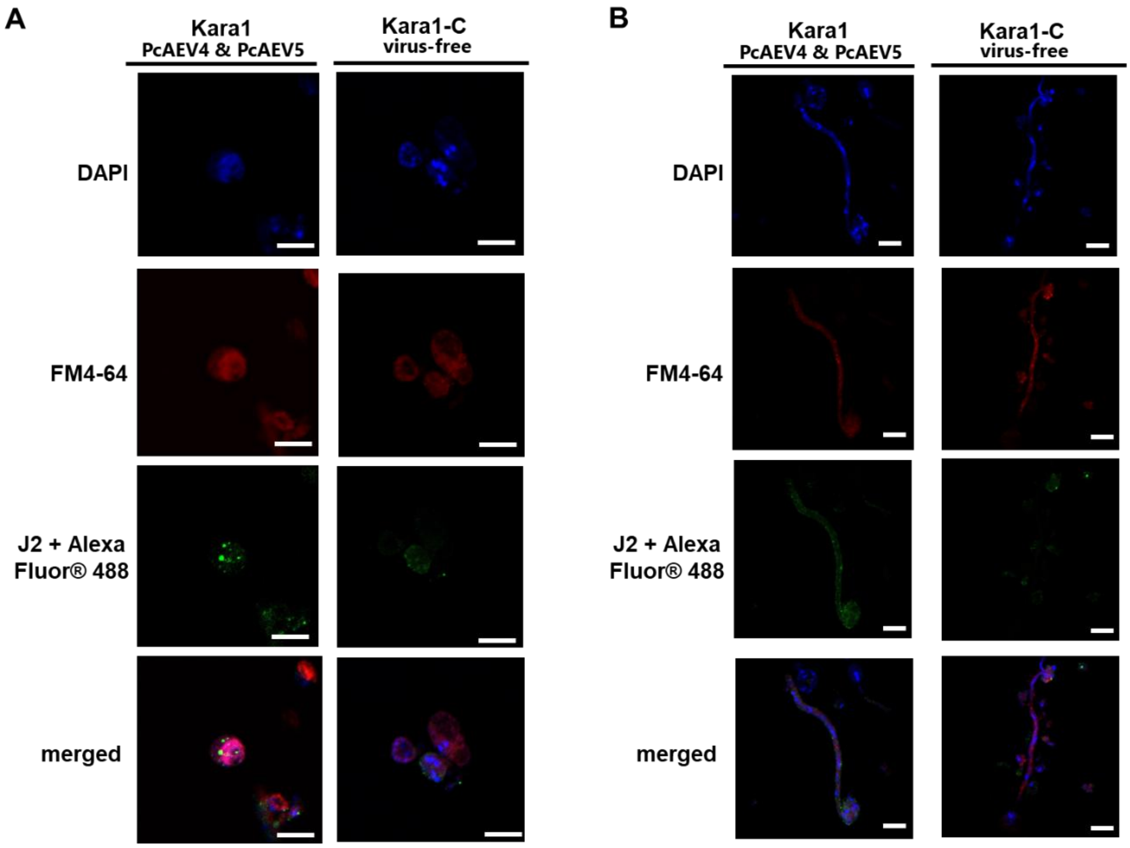

Supplemental Figure S5. Imaging analyses with a confocal laser scanning microscope (LSM710NLO; Carl Zeiss, Jena, Germany) were performed. (A) IFA and confocal laser microscopy with DAPI, FM4-64, and J2 antibodies in regenerating protoplasts of Kara1 and Kara1-C strains; Kara1-C was used as a negative control. **(B)** IFA and confocal laser microscopy observations in protoplast regenerated mycelia. Scale bar = 10 μm.
